# Prevalence of hepatitis B virus infection among Iranian high risk groups: a systematic review and meta-analysis

**Published:** 2018

**Authors:** Amir Almasi-Hashiani, Erfan Ayubi, Kamyar Mansori, Mostafa Salehi-Vaziri, Yousef Moradi, Behzad Gholamaliei, Salman Khazaei

**Affiliations:** 1 *Department of Epidemiology and Reproductive Health, Reproductive Epidemiology Research Center, Royan Institute for Reproductive Biomedicine, ACECR, Tehran, Iran*; 2 *Department of Epidemiology, School of Health, Arak University of Medical Sciences, Arak, Iran*; 3 *Department of Community Medicine, School of Medicine, Zahedan University of Medical sciences, Zahedan, Iran*; 4 *Health promotion research center, Zahedan University of Medical Sciences, Zahedan, Iran. *; 5 *School of Public Health, Dezful University of Medical Sciences, Dezful, Iran.*; 6 *Department of Epidemiology, School of Public Health, Iran University of Medical Sciences, Tehran, Iran*; 7 *Department of Arboviruses and Viral Hemorrhagic Fevers, Pasteur Institute of Iran, Tehran, Iran*; 8 *Pars Advanced and Minimally Invasive Medical Manners Research Center, Pars Hospital, Tehran, Iran*; 9 *Department of Health Education, School of Public Health, Hamadan University of Medical Sciences, Hamadan, Iran *; 10 *Department of Epidemiology, School of Public Health, Hamadan University of Medical Sciences, Hamadan, Iran. *; 11 *Department of Epidemiology and Biostatistics, School of Public Health, Tehran University of Medical Sciences, Tehran, Iran *

**Keywords:** Hepatitis B, Prevalence, Drug users, Sex workers, Prisoners, Meta-analysis, Iran

## Abstract

**Aim:**

Present study aimed to systematically review and quantitatively synthesize published data about the prevalence of Hepatitis B Virus (HBV) infection among high risk groups in Iran.

**Background:**

Determining true burden of Hepatitis B Virus (HBV) infection among high-risk groups relies on knowledge of occurrence measures such as prevalence rate. There is no conclusive and comprehensive data regarding to prevalence of HBV infection among high risk groups in Iran.

**Methods:**

Relevant studies were searched in PubMed, Scopus, Web of Knowledge and local databases. In addition, reference lists of relevant studies were searched manually. Two independent authors reviewed the eligibility of retrieved studies and extracted the required data. Studies reporting HBV infection among high risk groups were included in the meta-analysis using random effects models. Meta regression and sub-group analysis were considered as additional analyses.

**Results:**

The initial search yielded 566 citations. After the primary screen, 37 studies were selected for review. Meta-analysis results showed that pooled prevalence of HBV infection among high risk groups in Iran was 4.8% (95% confidence interval: 3.6%-6.1%), with the highest prevalence among in prisoners (5%; 3%-6%), and in central regions of Iran (7%; 4%-11%). Year of study may affect the observed heterogeneity in the estimated prevalence of HBV infection among injection drug users (IDUs) and prisoners.

**Conclusion:**

Our results indicate that prevalence of HBV infection among high risk groups was seemingly high in Iran. Health policy decision makers should be aware of prevalence of HBV infection among different high risk groups and in different regions of Iran.

## Introduction

 Chronic hepatitis B Virus (HBV) infection is defined as either existence of HBsAg in someone's blood who tests negative for IgM antibodies against hepatitis B core antigen (IgM anti-HBc), or existence of HBsAg in blood serum for at least 6 months (1). Despite availability of an efficient vaccine and powerful antiviral treatments, chronic HBV infection has remained one the most important public health concerns and a major cause of deaths from cirrhosis and liver malignancy worldwide (2-6). HBV is highly contagious and transmitted mainly via blood transfusion, unsafe injection practices, sexual contact, and mother-to-child transmission (7).

An important international study showed that global HBsAg sero-prevalence is 3.61%, with the highest prevalence rate in African countries (8.83%) and Western Pacific region (5.26%), respectively. This study showed that nearly 248 million people were HBsAg positive in 2010 (3). In a meta-analysis in 2016, the pooled prevalence of HBV infection among general population in Iran was 2.2% (5).

Global Burden of Disease, Mortality, and Causes of Death Collaborators estimate that HBV is one of first causes of mortality worldwide, accounting for 686,000 (520,000-866000) deaths annually. Age standardized death rate of HBV equals to 1.1 (95% CI: 0.8-1.3) case per 100,000 persons (6).

HBV is transmitted mainly by percutaneous or mucosal contact with infected blood or other body fluids (2). Therefore, some specific populations including prison inmates, injection drug users (IDUs), addict persons, and female sex workers (FSWs) are at higher risk of HBV infection (8-11). Consequently, having information about prevalence of HBV among high risk groups has been a concern to public health policy- makers and service providers. Policy-makers can use this information to effectively allocate resources and healthcare activities to truly needy population groups. On the other hand, accurate data about prevalence of HBV infection among different sub-groups of population, especially high risk groups, is vital to evaluate impacts of prevention programs, including vaccination and educational programs, and also to determine the burden of the disease. Although many studies have been carried out about HBV sero-prevalence in other countries, there is no comprehensive data regarding prevalence of HBV infection among Iranian high risk groups. Therefore, this study aimed to determine prevalence of HBV infection among Iranian high risk groups by conducting an up-to-date systematic review and meta-analysis. 

## Methods

**Search Strategy**

We utilized PRISMA statement as a guide to enhance quality of the review (12). Major international electronic bibliographic databases, including PubMed, Scopus and Web of Science and National databases including Magiran, Iranmedex, and SID, were searched from 2000 to 2016. In addition, reference lists of relevant studies were searched manually.

The following terms were used to search the international databases; ‘‘prostitute’’; ‘‘FSW’’ or ‘‘sex worker’’; ‘‘intravenous drug users’’; ‘‘drug addicts’’; ‘‘IDU’’ or ‘‘injection drug users’’; ‘‘prisoner’’; ‘‘jail’’; ‘‘inmate’’ or ‘‘prison’’; and ‘‘HBV’’ or ‘‘Hepatitis B’’ or ‘‘blood borne infection’’ and ‘‘Iran’’. The searching was restricted to studies in English conducted since 2000 and afterwards. 

Sex work was defined as exchange of sex for money, drugs, or goods (13). Also, IDUs were defined as people who inject narcotic substances into the body with a hollow needle and a syringe which is pierced through the skin into the body usually intravenously (14). Only the articles that determined HBV infection in patients by laboratory criteria, according to national guidelines, were included into the review (4). 

**Data Extraction and Quality Assessment**

Two independent authors (YM and KM) reviewed the retrieved studies from initial search by Title/ Abstract. For the studies that was eligible for meta-analysis, the following information were extracted: (1) name of the first author, (2) publication year and location of study conduction, (3) total sample size, (4) the reported prevalence of infection, (5) recruitment setting, (6) recruitment method, (7) age group and (7) high risk group (IDUs, FSWs, or prisoners). The kappa statistics (95%) was used to identify the inter-authors reliability. The third author (AA) was considered as arbiter to resolve any disagreements. The STROBE statement was used to assess the quality of studies.

**Statistical analysis**

At first, the variance of each study was calculated through the variance of a binomial distribution, given that the prevalence rate has a binomial distribution. Then, each study was given a weight, which was inversely proportional to the variance. 

The heterogeneity of results across studies was controlled for using chi-square (Chi2) test (with P-value <0.10) and I^2^ statistic. The I^2^ statistic greater than 75% was considered as indicator of significant heterogeneity across studies. Subgroups analysis was conducted on the basis of infection type, high risk group, and geographical regions. Meta regression was used to examine the impact of year of study on prevalence rate of HBV among prisoners and IDUs.

Random-effects meta-analysis model was used to pool the estimated prevalence of HBV from retrieved studies. All the analyses were performed using Stata software version 12 (Stata Corp, College Station, TX, USA) by the “metan”(15), “metareg”(16), “metabias”(17), “metacum”(18) and “metainf” (19) commands.

**Figure 1:**
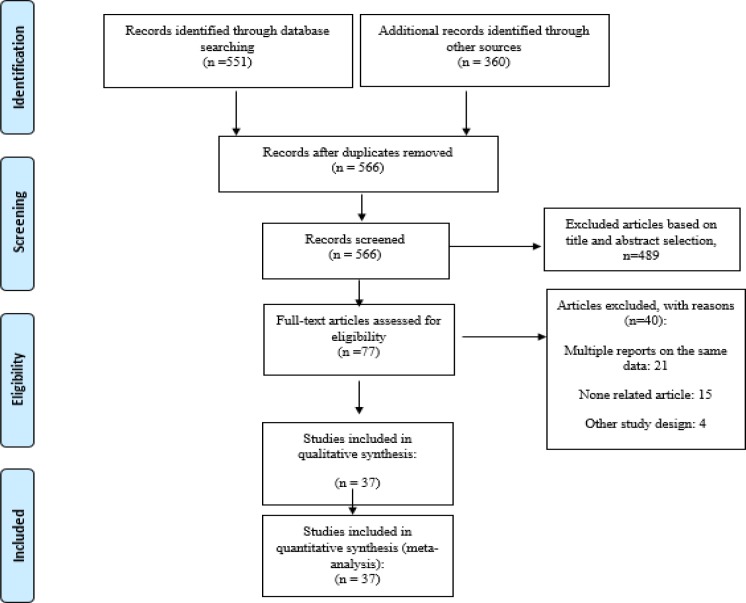
Flow diagram of the literature search for studies included in meta-analysis

## Results

Figure 1 shows the results of the literature review and selection process. A total of 911 potentially relevant articles were identified from the initial literature review. After removing duplicates, 566 articles remained and then the authors excluded other 489 articles by screening the titles and abstracts. Finally 37studies met eligibility criteria for meta-analysis that included 22057 HBV positive patients.

**Study Characteristics**

Full characteristics of primary included studies are shown in Table 1. These studies were published between 2003 and 2015. The sample size of included articles varied from 60 to 8630, with a total of 22057 cases with high risk behaviors, including FWS (2 studies), IDUs (19 studies), prisoners (13 studies) and drug addicts (2 studies). The lowest prevalence rate was reported by Teimori *et al.* in Kermanshah among IDUs (0 %) and the highest prevalence rate was reported by Amin-Esmaeili *et al*. among IDUs in Tehran (24.7%). 

**Evaluation of Heterogeneity and Meta-Analysis**

Results of Cochran’s Q test and I^2^ statistics indicated of significant heterogeneity among the included studies (Q=496.13, df =36, p<0.001 and I^2^=92.74%). The pooled prevalence of HBV infection in high risk groups was 4.8% (95% CI: 3.6%-6.1%). In order to reduce the heterogeneity, the authors conducted a subgroup analysis based on geographical regions (Table 2). Accordingly, the prevalence of HBV among high risk groups in north, west, southwest, east, northeast, and central regions of Iran was 7 (95% CI: 4.0-11.0), 3 (95% CI: 1.0, 6.0), 5 (95% CI: 3.0-7.0) and 5% (95% CI: 3.1, 7.3), respectively.

**Meta Regression**

Results of meta-regression analysis are shown in Fig 4 and 5. According to the findings, prevalence of HBsAg among prisoners was not related to year of study and its decreasing trend over the years was not significant (*P*=0.72). In addition, increasing trend of HBV prevalence among IDUs over the years was not significant also (*P*=0.4).

**Sensitivity Analysis**

Sensitivity analysis showed that exclusion of individual studies could not substantially change the results. The pooled prevalence of HBsAg positive ranged from 4.47%, 95% CI: 3.12-5.8 (when excluding Mardani *et al. *(21) to 5.97%, 95% CI: 4.28-7.66 (when excluding Soud bakhsh *et al*. (41).

**Cumulative Meta-Analysis**

**Figure 2:**
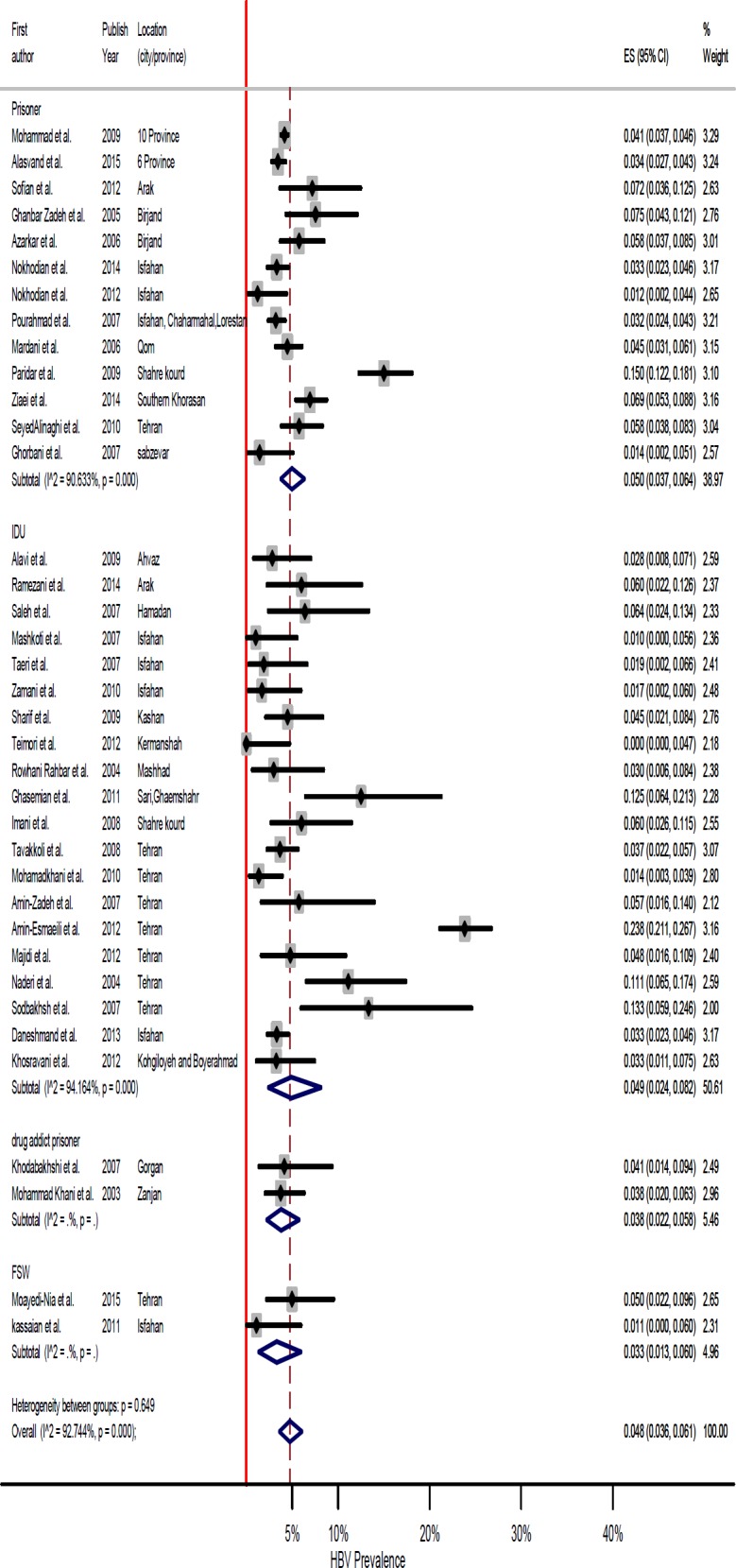
Forest plot of prevalence of HBV infection in high risk groups in Iran

As shown in figure 2, cumulative meta-analysis of HBs Ag positive based on publication year, revealed that the overall prevalence estimates were constant in high risk groups and that the 95% CIs narrowed with accumulation of primary study data over time.

## Discussion

Despite availability of an effective vaccine against hepatitis B since early 1990s, covering more than 400 million people, the disease remains a major public health problem throughout the world (54). People at elevated risk of HBV infection are a key group that should be specifically targeted for prevention and control measures (55). For such attempts, to be properly scaled, targeted, having updated, detailed, and accurate data about the high risk populations is a prerequisite. 

Our systematic review showed that the overall prevalence of HBV infection among high-risk groups in Iran is nearly 4.8%, with clear geographical differences across the country. More specifically, the prevalence of HCV infection was the highest in northern parts of the country (prevalence proportion of 7% in Gorgan, Sari and Ghaemshahr). On the other hand, this proportion was lowest in west and southwest of Iran (prevalence proportion of 3% in Hamadan, Kermanshah, Zanjan, and Ahvaz). As well, results showed that year of study can lead to heterogeneity in estimated prevalence of HBV infection IDUs and prisoners. 

**Table 1:** Description of the studies included in the meta-analysis

First author/year (Ref)	Sample size	Location	High risk group	Recruitment setting	Recruitment method	Age group	Gender
Ghasemian et al. (2011) (20)	88	Sari, Ghaemshahr	IDU	Hospital	Census	35.01±11.53	Both
Ziaei et al. (2014) (8)	881	Southern Khorasan	Prisoner	Prison	random sampling	34.7 ±11.4	Both
Mardani et al. (2006) (21)	808	Qom	Prisoner	Prison	Census	Mean=35	Both
Naderi et al. (2004) (22)	144	Tehran	IDU	Hospital	Census	26-30	Both
Zamani et al. (2010) (23)	118	Isfahan	IDU	Community	peer-driven sampling	29.0 ±6.6	Both
Paridar et al. (2009) (24)	600	Shahr kord	Prisoner	Prison	Census	NA	Both
Amin-Esmaeili et al. (2012) (25)	899	Tehran	IDU	drug treatment centers	purposive sampling	33.9 ±9.4	Both
Saleh et al. (2007) (26)	94	Hamadan	IDU	Forensic medicine	Census	NA	Both
Khodabakhshi et al. (2007) (27)	121	Gorgan	drug addict prisoner	Prison	random sampling	NA	Both
Mohamadkhani et al. (2010) (28)	220	Tehran	IDU	MMT center	Census	NA	Both
Tavakkoli et al. (2008) (29)	518	Tehran	IDU	Combined	volunteers	NA	Both
Azarkar et al. (2006) (30)	400	Birjand	Prisoner	Prison	Stratified random sampling	34.1±11.7	Both
Sharif et al. (2009) (31)	200	Kashan	IDU	Hospitalized	volunteers	36.5 ± 10.2	Both
Alavi et al. (2010) (32)	142	Ahvaz	IDU	Hospitalized	NA	26.3±5.7	Both
Majidi et al. (2012) (33)	104	Tehran	IDU	Hospital	NA	NA	Both
Ghorbani et al. (2007) (34)	139	Sabzevar	Prisoner	prison	NA	NA	Both
Asghari et al. (2009) (35)	8630	10 Province	Prisoner	Prison	volunteers	NA	Both
Imani et al. (2008) (36)	133	Shahre kourd	IDU	voluntary rehabilitation center	Census	31.3±7.1	Both
Ghanbarzadeh et al. (2006) (37)	199	Birjand	Prisoner	Prison	volunteers	35±12.3	Female
Teimori et al. (2012) (38)	76	Kermanshah	IDU	MMT center	Convenience sampling	35.2±0.99	Female
Nokhodian et al. (2012) (39)	161	Isfahan	Prisoner	Prison	Census	34.54 ± 11.2	Female
Moayedi-Nia et al. (2015) (40)	161	Tehran	FSW	NA	respondent driven sampling	37.43 ± 8.9	Female
Soudbakhsh et al. (2007) (41)	60	Tehran	IDU	Hospital	Census	35.3±9.68	Male
Taeri et al. (2007) (42)	106	Isfahan	IDU	Hospital	volunteers	50.8±8.1	Male
Amin-Zadeh et al. (2007) (43)	70	Tehran	IDU	Hospital	volunteers	34.4±9.6	Male
Alasvand et al. (2015) (44)	2120	6 Province	Prisoner	prison	random sampling	37±13	Male
Rowhani Rahbar et al. (2004) (45)	101	Mashhad	IDU	Prison	convenience sample	32.8	Male
SeyedAlinaghi et al. (2010) (46)	452	Tehran	Prisoner	Prison	volunteers	NA	Male
Ramezani et al. (2014) (9)	100	Arak	IDU	MMT center	Census	17-58	Male
Pourahmad et al. (2007) (47)	1431	Isfahan, Lorestan Chaharmahal,	Prisoner	Prison	NA	25-60	Male
Sofian et al. (2012) (48)	153	Arak	Prisoner	Prison	Census	30.7 ±5.9	Male
Nokhodian et al. (2014) (10)	970	Isfahan	Prisoner	Prison	volunteers	32.61 ± 8.1	Male
Meshkati et al. (2007) (49)	98	Isfahan	IDU	Behavioral consulting canter	Census	30- 40	Male
Khani et al. (2003) (50)	346	Zanjan	drug addict prisoner	Prison	NA	33.7 ± 10.2	Male
Daneshmand et al. (2013) (51)	970	Isfahan	IDU	Prison	Census	36.6±0.31	Male
Khosravani et al. (2012) (52)	153	Kohgiloyeh & Boyerahmad	IDU	Community	Census	34.9	Both
Kassaian et al. (2011) (53)	91	Isfahan	FSW	DIC	Snowball sampling	30.84±9.34	Female

**Table 2:** Meta-analysis of prevalence of HBV infection based on high risk groups in different geographic area of Iran

Geographic location	N of study (sample size)	Prisoners prevalence (%)	IDUs prevalence (%)	FSWs prevalence (%)	Drug addict prisoners prevalence (%)	Overall prevalence (%)
2 (209)	-	13 (6, 21)	-		4 (1, 9)	7 (4, 11)
4 (658)	-	2 (0, 7)	-		4 (2, 6)	3 (1, 6)
5 (1841)	5 (3, 8)	3 (1, 8)	-		-	5 (3, 7)
24 (8720)	5.2 (2.7, 8.3)	5.2 (2.2, 9.3)	3.3 (1.3, 6)		-	5 (3.1, 7.3)

**Figure 3:**
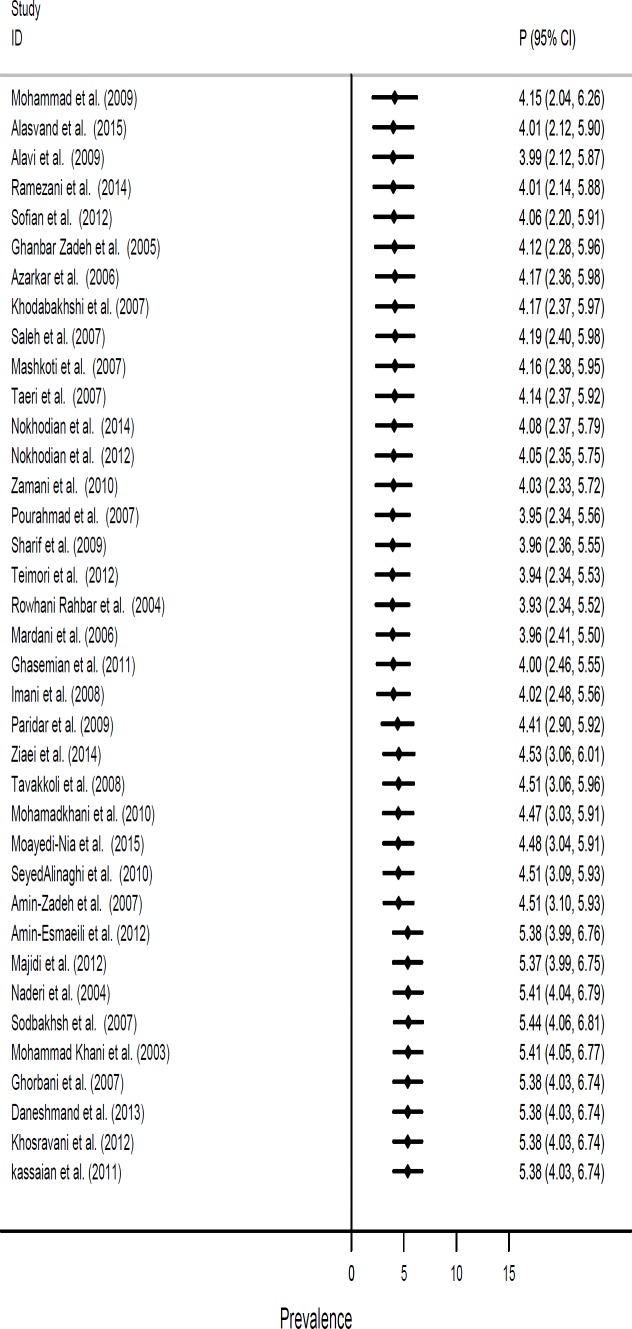
Forest plot showing cumulative prevalence of HBV Infection in Iranian high risk groups according to time

**Figure 4.**
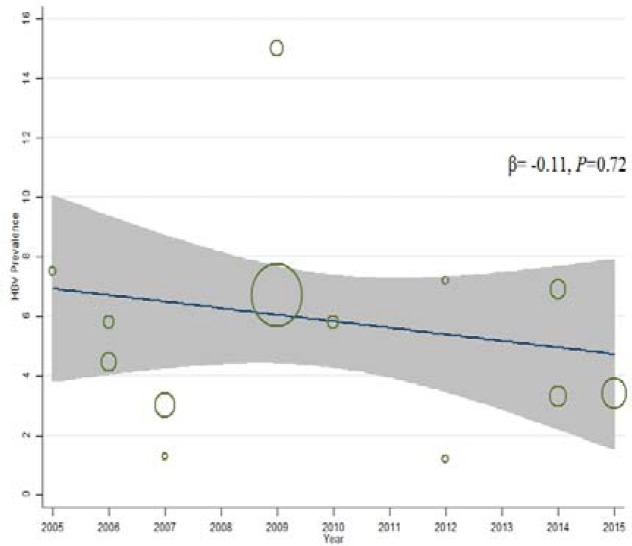
Meta-regression plots of change in prevalence of HBV among prisoners according years of study

**Figure 5:**
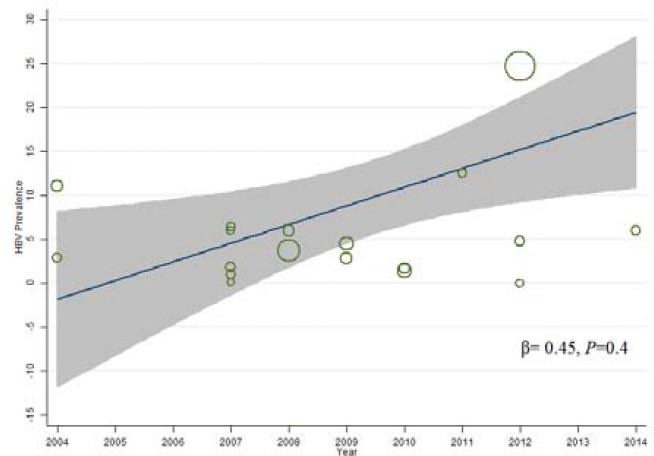
Meta-regression plots of change in prevalence of HBV among IDUs according years of study

In 2009, Poorolajal *et al*. (56), reported of an estimated rate of 3.2% HBV infection among Iranian high risk groups. However their review covered only 4 studies (8, 29, 57, 58) on IDUs from 2001 to 2003. Unfortunately, there has been no comprehensive systematic review of HBV prevalence in high risk population in Iran since then. To estimate HBV infection prevalence among Iranian high risk groups, an extensive literature review spanning from 2003 to 2015 was carried out in the present study. The results of our systematic review revealed that the estimated prevalence of HBV infection among Iranian high risk population was 4.8 % (95% CI: 3.6%-6.1%), a rate which is over two times higher than HBV prevalence among the general population of Iran (2.2%) (5). 

Significant reduction in prevalence of HBV infection is expected to occur with implementation of national vaccination programs and other preventive measures. A recent systematic review by Salehi-Vaziri *et al.* (5) revealed a substantial reduction of HBV infection among general population of Iran. The estimated rate of HBV infection before 2010 was 2.9%, while the rate was 1.3% in 2010 and onwards (5). In the present study, however the cumulative meta-analysis revealed that the prevalence of HBV infection was constant over the time among Iranian high risk groups.

This observation can be explained by two reasons: 1) the fact that the majority of study population was born before 1993, the year when infant HBV vaccination was launched in the country, and 2) potential low efficacy of HBV immunization, in case if they received HBV vaccine, which has been documented by several reports (59-63).

Our review revealed a significant heterogeneity in prevalence of HBV infection among high risk groups from different geographical areas of Iran. Analysis of HBV prevalence within 4 geographical categories of north, west/southwest, east/northeast, and center showed the highest prevalence in north (7%) and the lowest in west/southwest regions (3%). The heterogeneity in geographical distribution of HBV infection was also indicated by two previous systematic reviews on general population in Iran. Therefore, it could be suggested that risk factors of HBV infections may be different across various regions of Iran and further studies on social, cultural, structural and behavioral factors affecting HBV infection in different parts of the country are clearly needed (5, 64). Nevertheless, there is a paucity of knowledge about the prevalence of HBV in many provinces of Iran, as the studies included in the present review hardly covered half of all provinces of Iran. To fill this gap, an active surveillance system should be established to monitor the burden of HBV among high risk groups throughout the country. 

Given that HBV is a sexually transmitted infection, sex workers are at elevated risk of contracting the virus. It is estimated that sexual contact is the mode of infection transmission in nearly 50% of HBV cases among American young adults (65). However, there is a huge lack of knowledge not only about the rate of HBV infection, but also on other sexually transmitted infections (STIs) in Iran (66). This review highlights the lack of enough knowledge on prevalence of HBV among sex workers in Iran. Given that implementation of educational programs about sexual issues cannot be scaled up in Iran, due to some specific cultural features of Iranian society (66), monitoring of this high risk group is a matter of great importance in order to decrease the burden of HBV infection. 

Previous reviews on general population of Iran indicated higher prevalence rate of HBV infection among men (5, 64). However, the authors were not able to estimate the prevalence of the infection based on gender in the present study, as almost all publications included in the current review were conducted exclusively on men. Although risk factors are more common in men, women should also be evaluated in terms of HBV infection and its risk factors in the country, especially in light of the fact that the number of women with high risk activities is increasing in Iran. 

Our results indicate that prevalence of HBV infection among high risk groups was seemingly high in Iran. Health policy decision-makers should be aware of prevalence of HBV infection among different high risk groups and in different regions of Iran.

## References

[1] Shepard CW, Simard EP, Finelli L, Fiore AE, Bell BP (2006). Hepatitis B virus infection: epidemiology and vaccination. Epidemiol Rev.

[2] Ott J, Stevens G, Groeger J, Wiersma S (2012). Global epidemiology of hepatitis B virus infection: new estimates of age-specific HBsAg seroprevalence and endemicity. Vaccine.

[3] Schweitzer A, Horn J, Mikolajczyk RT, Krause G, Ott JJ (2015). Estimations of worldwide prevalence of chronic hepatitis B virus infection: a systematic review of data published between 1965 and 2013. Lancet.

[4] Beasley RP (2009). Rocks along the road to the control of HBV and HCC. Ann Epidemiol.

[5] Salehi-Vaziri M, Sadeghi F, Hashiani AA, Fesharaki MG, Alavian SM (2016). Hepatitis B virus infection in the general population of Iran: an updated systematic review and meta-analysis. Hepat mon.

[6] Liang TJ (2009). Hepatitis B: the virus and disease. Hepatology.

[7] Lok AS, McMahon BJ (2007). Chronic hepatitis B. Hepatol.

[8] Ziaee M, Sharifzadeh G, Namaee MH, Fereidouni M (2014). Prevalence of HIV and hepatitis B, C, D infections and their associated risk factors among prisoners in Southern Khorasan Province, Iran. Iran J Public Health.

[9] Ramezani A, Amirmoezi R, Volk JE, Aghakhani A, Zarinfar N, McFarland W (2014). HCV, HBV, and HIV seroprevalence, coinfections, and related behaviors among male injection drug users in Arak, Iran. AIDS Care.

[10] Nokhodian Z, Yaran M, Adibi P, Kassaian N, Meshkati M, Ataei B (2014). Seroprevalence of hepatitis B markers among incarcerated intravenous drug users. J Res Med Sci.

[11] Amin-Esmaeili M, Rahimi-Movaghar A, Razaghi EM, Baghestani AR, Jafari S (2012). Factors correlated with hepatitis C and B virus infections among injecting drug users in Tehran, IR Iran. Hepat Mon.

[12] Moher D, Liberati A, Tetzlaff J, Altman DG (2009). Preferred reporting items for systematic reviews and meta-analyses: the PRISMA statement. Ann intern med.

[13] Alter MJ (2006). Epidemiology of viral hepatitis and HIV co-infection. J hepatol.

[14] WHO Hepatitis B Fact sheet. 2016. http://www.who.int/mediacentre/factsheets/fs204/en/.

[15] Harris R, Bradburn M, Deeks J, Harbord R, Altman D, Steichen T (2010). METAN: Stata module for fixed and random effects meta-analysis. Statistical Software Components.

[16] Harbord RM, Higgins J (2008). Meta-regression in Stata. Meta.

[17] Goodman S, Dickersin K (2011). Metabias: A challenge for comparative effectiveness research. Ann intern med.

[18] Bradburn MJ, Deeks J, Altman D (2004). Updated and new commands for meta-analysis in STATA. Cancer Research UK Medical Statistics Group Oxford: Centre for Statistics in Medicine.

[19] Steichen T (2001). METANINF: Stata module to evaluate influence of a single study in meta-analysis estimation. Statistical Software Components.

[20] Ghasemian R, Najafi N, Amirkhanloo K (2011). The study of infections due to injection drug abuse in the injecting drug users hospitalized at imam khomeini hospital in Sari and Razi Hospital in Ghaemshahr in 2007-2009. J Mazand Univ Med Sci.

[21] Mardani A, Shahsavarani M, Zibafar MS, Mardani H, Gonlouei SH, Rahchamandi Z (2009). Seroprevalence of Hepatitis B Virus Surface Antigen (HBsAg) in Addict Prisoners of Central Prison of Qom Province during 2004-2005. Iran j inf dis trop med.

[22] Naderi N Epidemiology of hepatitis C in Hospitalized Addicts in in Loghman Hakim Hospital, 2000-2001 [MD Thesis]. Tehran: Shahid Beheshti University of Medical Sciences.

[23] Zamani S, Radfar R, Nematollahi P, Fadaie R, Meshkati M, Mortazavi S (2010). Prevalence of HIV/HCV/HBV infections and drug-related risk behaviours amongst IDUs recruited through peer-driven sampling in Iran. Int J Drug Policy.

[24] Paridar F, Tajbakhsh E (2008). Seroprevalence of HBV infection in Shahrekord prisoners via EIA. World microbes J.

[25] Amin-Esmaeili M, Rahimi-Movaghar A, Razaghi EM, Baghestani AR, Jafari S (2012). Factors Correlated With Hepatitis C and B Virus Infections Among Injecting Drug Users in Tehran, IR Iran. Hepat Mon.

[26] Saleh M, Kazemifar AM, Saleh AE, Asghar A, Nobari H, Samimi R (2010). Prevalence of HIV, Hepatitis B and C Sero-positivity in Expired IV Drug Abusers in Hamedan. Forensic Medicine J.

[27] Khodabakhshi B, Abbassi A, Fadaee F, Rabiee M (2007). Prevalence and Risk Factors of HIV, Hepatitis B Virus and Hepatitis C Virus Infections in Drug Addicts among Gorgan Prisoners. J Med Sci.

[28] Mohammadkhani-Ghiasvand A, Golian-Tehrani S, Modanlu S, Vejdani M, Babaei-Heydarabadi A, Dehghankar L (2016). Investigation of Serologic Prevalence of HIV and Hepatitis B Infections and its Relationship with Behavioral Risk Factors among Drug Addicts. Journal of Health System Research.

[29] Tavakkoli H, Mir-Nasseri MM, Poustchi H, Afshar P, Motalebi MN, Mohammadkhani A (2008). Prevalence and Risk Factors of Hepatitis B Infection in Injection Drug Users, Tehran (2001-2002). Hepat Mon.

[30] Azarkar Z, Sharifzadeh G, Miraki M (2007). HBV, HCV and HIV prevalence among South Khorasan prisoners. Scientific Journal of Birjand University of Medical Sciences.

[31] Sharif M, Sherif A, Sayyah M (2009). Frequency of HBV, HCV and HIV infections among hospitalized injecting drug users in Kashan. Indian J Sex Transm Dis.

[32] Alavi SM, Behdad F (2010). Seroprevalence study of hepatitis C and hepatitis B virus among hospitalized intravenous drug users in Ahvaz, Iran (2002-2006). Hepat Mon.

[33] Majidi M (2012). Prevalence of HIV, hepatitis B and C among drug abusers admitted to ICU of Baharloo hospital in 2010 [PhD Thesis of Forensic Medicine].

[34] Ghorbani A, Masoudifar M, Tasbandi R, Mirmousavi SJ, Ebadollahzadeh H (2007). Prevalence of hepatitis B in the city of Sabzevar prisoners from 2004 to 2006.

[35] Asgari F, Gouya MM, Mohammad K, Fotouhi A, Yousefi A (2008). Hepatitis C virus infection among Iranian prisoners and its relation with addiction, 2001-2005. Hakim.

[36] Imani R, Karimi A, Rouzbahani R, Rouzbahani A (2008). Seroprevalence of HBV, HCV and HIV infection among intravenous drug users in Shahr-e-Kord, Islamic Republic of Iran. East Mediterr Health J.

[37] Ghanbarzadeh N, Najafi-Semnani M (2006). A study of HIV and other sexually transmited infections among female prisoners in Birjand. Scientific Journal of Birjand University of Medical Sciences.

[38] Teimouri F, Kariman N, Mansouri F, Rezaei M (2011). The prevalence of high-risk behavior and STIs in women referred to Niloofar addiction treatment centers in Kermanshah, 2009-2010. Behbood.

[39] Nokhodian Z, Yazdani MR, Yaran M, Shoaei P, Mirian M, Ataei B (2012). Prevalence and risk factors of HIV, syphilis, hepatitis B and C among female prisoners in Isfahan, Iran. Hepat Mon.

[40] Moayedi-Nia S, Jozani ZB, Djavid GE, Entekhabi F, Bayanolhagh S, Saatian M (2016). HIV, HCV, HBV, HSV, and syphilis prevalence among female sex workers in Tehran, Iran, by using respondent-driven sampling. Aids Care.

[41] Soudbakhsh A, Nami M, Hadjiabdolbaghi M, Kazemi B (2008). Transfusion Transmitted Virus prevalence rate in Injection Drug Users (IDUs): a cross sectional study. Tehran University Medical Journal.

[42] Talaie H, Shadnia SH, Okazi A, Pajouhmand A, Hasanian H, Arianpoor H (2007). The prevalence of hepatitis B, hepatitis C and HIV infections in non-IV drug opioid poisoned patients in Tehran-Iran. Pak J Biol Sci.

[43] Aminzadeh Z, Sarhangi-poor KA (2007). Sero epidemiology of HIV, HBV, HCV and Syphilis among hospitalized IDUs in Loghman-Hakim hospital. Iranian Journal of Medical Microbiology.

[44] Alasvand R, Azimian F, Nabavi M, Hosseini-Zijood M (2015). The prevalence of hepatitis B and C in male prisoners in Iranian prisons in 2011. Pajoohandeh.

[45] Rowhani-Rahbar A, Rooholamini S, Khoshnood K (2004). Prevalence of HIV infection and other blood-borne infections in incarcerated and non-incarcerated injection drug users (IDUs) in Mashhad, Iran. Int J Drug Policy.

[46] SeyedAlinaghi SA, Kheirandish P, Karami N, Salem S, Shirzad H, Jahani MR (2010). High prevalence of chronic hepatitis B infection among injection drug users in Iran: the need to increase vaccination of adults at risk. Acta Med Iran.

[47] Pourahmad M, Javady A, Karimi I, Ataei B, Kassaeian N (2007). Seroprevalence of and risk factors associated with hepatitis B, hepatitis C, and human immunodeficiency virus among prisoners in Iran. Infect Dis Clin Pract.

[48] Sofian M, Aghakhani A, Banifazl M, Azadmanesh K, Farazi AA, McFarland W (2012). Viral hepatitis and HIV infection among injection drug users in a central iranian city. J Addict Med.

[49] Meshkati M, Taeri K, Etedali E, Farid F (2007). Prevalence of Hepatitis B, C and HIV/AIDS in injecting drug users referred to the Behavioral Diseases Counseling Center in 2004.

[50] Khani M, Vakili M-M (2003). Prevalence and risk factors of HIV, Hepatitis B virus and Hepatitis C virus infections in drug addicts among Zanjan Prisoners. Arch Iranian Med.

[51] Daneshmand D, Nokhodian Z, Adibi P, Ataei B (2013). Risk Prison and Hepatitis B Virus Infection among Inmates with History of Drug Injection in Isfahan, Iran. Scientific World J.

[52] Khosravani A, Sarkari B, Negahban H, Sharifi A, Toori MA, Eilami O (2012). Hepatitis B Infection among high risk population: a seroepidemiological survey in Southwest of Iran. BMC Infect Dis.

[53] Kassaian N, Ataei B, Yaran M, Babak A, Shoaei P (2011). Hepatitis B and C among women with illegal social behavior in Isfahan, Iran: Seroprevalence and associated factors. Hepat Mon.

[54] Venook AP, Papandreou C, Furuse J, de Guevara LL (2010). The incidence and epidemiology of hepatocellular carcinoma: a global and regional perspective. Oncologist.

[55] Nelson PK, Mathers BM, Cowie B, Hagan H, Des Jarlais D, Horyniak D (2011). Global epidemiology of hepatitis B and hepatitis C in people who inject drugs: results of systematic reviews. Lancet.

[56] Poorolajal J, Majdzadeh R (2009). Prevalence of chronic hepatitis B infection in Iran: a review article. J Res Med Sci.

[57] Jahani MR, Alavian SM, Shirzad H, Kabir A, Hajarizadeh B (2005). Distribution and risk factors of hepatitis B, hepatitis C, and HIV infection in a female population with "illegal social behaviour". Sex Transm Infect.

[58] Behnaz K, Abdollah A, Fateme F, Mohammadreza R (2007). Prevalence and risk factors of HIV, hepatitis B virus and hepatitis C virus infections in drug addicts among Gorgan prisoners. J Med Sci.

[59] Al Ghamdi SS, Fallatah HI, Fetyani DM, Al-Mughales JA, Gelaidan AT (2013). Long-term efficacy of the hepatitis B vaccine in a high-risk group. J Med Virol.

[60] Elrashidy H, El-Didamony G, Elbahrawy A, Hashim A, Alashker A, Morsy MH (2014). Absence of occult hepatitis B virus infection in sera of diabetic children and adolescents following hepatitis B vaccination. Hum Vaccin Immunother.

[61] Masuet-Aumatell C, Ramon-Torrell JM, Casanova- Rituerto A, Banque-Navarro M, Davalos-Gamboa Mdel R, Rodriguez SL (2013). Seroprevalence of hepatitis B in two period birth cohorts of Bolivian children: effect of universal vaccination. Trans R Soc Trop Med Hyg.

[62] Mizusawa M, Perlman DC, Lucido D, Salomon N (2014). Rapid loss of vaccine-acquired hepatitis B surface antibody after three doses of hepatitis B vaccination in HIV-infected persons. Int J STD AIDS.

[63] Nisihara R, De Bem RS, Negreiros PH, Utiyama SR, Oliveira NP, Amarante H (2014). Low hepatitis B vaccine response in children with Down syndrome from Brazil. Child Care Health Dev.

[64] Alavian SM, Hajarizadeh B, -Asl MA, Kabir A, Lankarani KB (2008). Hepatitis B Virus Infection in Iran: A Systematic Review. Hepat Mon.

[65] Atkins M, Nolan M (2005). Sexual transmission of hepatitis B. Curr Opin Infect Dis.

[66] Alavian SM, Fallahian F, Lankarani KB (2007). The changing epidemiology of viral hepatitis B in Iran. J Gastrointestint Liver Dis.

